# Assessing The Feasibility Of A Virtual Delivery Of The Savvy Caregiver Program For Jamaican Caregivers Of Persons Living With Dementia

**DOI:** 10.1002/alz70858_096614

**Published:** 2025-12-24

**Authors:** Glenna S Brewster, Janelle Robinson, Melinda Higgins, Catherine Brown, Jay‐Bethenny Gallimore, Jodi Sutherland Charvis, Jordan Pelkmans, Kenneth Hepburn, Ishtar Govia

**Affiliations:** ^1^ Emory University, Nell Hodgson Woodruff School of Nursing, Atlanta, GA, USA; ^2^ CUNY Graduate Center, New York, NY, USA; ^3^ Emory University Nell Hodgson Woodruff School of Nursing, Atlanta, GA, USA; ^4^ University of the West Indies, Bridgetown, St. Michael, Barbados; ^5^ Centre for Global Mental Health, Health Service and Population Research Department, Institute of Psychiatry, King's College London, London, England, United Kingdom; ^6^ University of Rhode Island, Kingston, RI, USA; ^7^ Emory University, Atlanta, GA, USA; ^8^ Amagi Health, London, England, United Kingdom

## Abstract

**Background:**

Psychoeducational interventions for caregivers of persons living with dementia (PLwD) are well‐documented in high‐income countries but are scarce in low‐ and middle‐income countries (LMICs). This study explored the feasibility of virtually delivering the Savvy Caregiver Program (SCP) to Jamaican caregivers of PLwD.

**Method:**

Thirty‐one caregivers in Jamaica participated in a virtual SCP intervention, consisting of 12 hours delivered over six weeks in two‐hour group sessions. Measures assessing perceived stress, mastery, depression, and burden were collected pre‐ and post‐intervention. Paired t‐tests were conducted to examine changes in caregiver outcomes.

**Result:**

Caregivers (mean age = 53.5 years) were predominantly female (87.1%) and children of PLwD (71.0%), while PLwD had a mean age of 80.2 years. Results showed a significant reduction in perceived stress scores (mean change = ‐3.32 ± 6.89, *p* = .017), with a moderate effect size (Cohen's *d* = 0.482). No significant changes were observed in caregiver mastery, burden, or depression.

**Conclusion:**

Virtual delivery of SCP moderately reduced perceived stress among Jamaican caregivers, demonstrating its feasibility and potential as a valuable intervention in LMIC settings. However, the lack of significant improvements in other caregiver outcomes highlights the need for culturally tailored interventions to address the broader needs of Jamaican caregivers. Further research with larger samples and longer follow‐up periods is warranted to examine long‐term benefits and enhancements of SCP in Jamaica and similar settings.

